# A Comprehensive Review of the Progress and Evaluation of the Thyroid Ultrasound Examination Program, the Fukushima Health Management Survey

**DOI:** 10.2188/jea.JE20210271

**Published:** 2022-12-05

**Authors:** Hiroki Shimura, Satoru Suzuki, Susumu Yokoya, Manabu Iwadate, Satoshi Suzuki, Takashi Matsuzuka, Noriko Setou, Tetsuya Ohira, Seiji Yasumura, Shinichi Suzuki, Hitoshi Ohto, Kenji Kamiya, Thyroid Ultrasound Examination Group

**Affiliations:** 1 Radiation Medical Science Center for the Fukushima Health Management Survey, Fukushima Medical University, Fukushima, Japan; 2 Department of Laboratory Medicine, Fukushima Medical University School of Medicine, Fukushima, Japan; 3 Thyroid and Endocrine Center, Fukushima Medical University, Fukushima, Japan; 4 Department of Thyroid and Endocrinology, Fukushima Medical University School of Medicine, Fukushima, Japan; 5 Department of Disaster Psychiatry, Fukushima Medical University School of Medicine, Fukushima, Japan; 6 Department of Epidemiology, Fukushima Medical University School of Medicine, Fukushima, Japan; 7 Department of Public Health, Fukushima Medical University School of Medicine, Fukushima, Japan; 8 Research Institute for Radiation Biology and Medicine, Hiroshima University, Hiroshima, Japan

**Keywords:** Fukushima, Thyroid Ultrasound Examination, children, ultrasonography, thyroid cancer

## Abstract

The Great East Japan Earthquake on March 11, 2011, and the subsequent tsunami caused an accident at the Fukushima Daiichi Nuclear Power Plant, in which extensive damage to the nuclear power reactors resulted in massive radioactive contamination. Fukushima Prefecture implemented the Thyroid Ultrasound Examination (TUE) program as part of the Fukushima Health Management Survey project in response to residents’ anxieties about health risks due to radiation exposure for residents aged 0–18 years at the time of the nuclear accident. This program consisted of the primary examination and the confirmatory examination. In the primary examination, thyroid nodules and cysts were examined using portable ultrasound apparatuses. The confirmatory examination was performed to have clinical or cytological diagnosis. As of June 30, 2021, 116, 71, 31, 36, and 9 examinees in the first, second, third, and fourth round of surveys, and the survey at age 25 years, respectively, were determined to have nodules cytologically diagnosed as malignant or suspicious for malignancy. The confirmatory examination of the fourth-round survey and the primary and confirmatory examination of fifth-round survey are currently in progress. Together with the low thyroid absorbed radiation dose estimated in the United Nations Scientific Committee on the Effects of Atomic Radiation 2020 report, our results suggested that the increased incidence of childhood thyroid cancer in Fukushima Prefecture was not caused by radiation exposure, but rather by the highly sensitive detection method. As detailed in this review, there were ongoing challenges in our program, such as actions against the risk of overdiagnosis and psychological support for participants and their families.

## INTRODUCTION

The Great East Japan Earthquake that occurred in 2011 caused a melt-through of the reactors at the Fukushima Daiichi Nuclear Power Plant, which are located on the Pacific coast of Fukushima Prefecture. In the nuclear accident at Chernobyl that occurred in 1986, there was internal exposure to radioactive iodine that induced thyroid cancer, especially in residents who were children at the time of accident.^1^ Therefore, there was concern about the health hazards due to radiation exposure from the radioactive materials released in Fukushima.^2^

Exposure of the thyroid gland to radiation causes thyroid cancer; however, the pathological or molecular biological differences between cancer caused by radiation and other cancer types are not definitively defined. Therefore, epidemiological analysis is considered to be the most effective method for assessing the effects of radiation on the risk of developing thyroid cancer. Before the earthquake, however, there was insufficient data on thyroid cancer in the pediatric population. Since there was a latent period of 4 to 5 years for the rapid increase in thyroid cancer in Chernobyl,^3^ commencing medical examinations immediately after the earthquake in Fukushima provided data for use as the basis for comparison.^2^^,^^4^ It was expected that the number of people who wished to undergo ultrasound examinations in Fukushima Prefecture would increase due to radiation anxiety, even if thyroid examinations were not performed. Therefore, it was important to carry out proper medical examinations. The Fukushima Prefectural Assembly also unanimously resolved to carry out thyroid examinations.

To monitor and support the health of Fukushima residents over a long-term period, the Fukushima Prefecture government initiated the Fukushima Health Management Survey.^2^^,^^4^^,^^5^ As one of these detailed surveys, the Thyroid Ultrasound Examination (TUE) program was planned. The diagnostic criteria and protocol were introduced and evaluated under an external committee of thyroid specialists in cooperation with associated academic societies.^4^ The criteria for the TUE program were developed based on the ultrasound diagnostic criteria for thyroid nodules published by the Japan Society of Ultrasonics in Medicine^6^ and the guideline for the implementation of fine needle aspiration cytology (FNAC) by the Japan Association of Breast and Thyroid Sonology (JABTS),^7^ both of which were developed prior to the disaster, in response to the need for precision management, and the countermeasures against overdiagnosis of low-risk thyroid cancer (eMaterial 1). After approval by the Prefectural Oversight Committee, the TUE program commenced in October 2011 for all residents aged 18 years or younger at the time of the earthquake who lived in Fukushima Prefecture.

## METHODS

### Program structure

**Figure 1.**
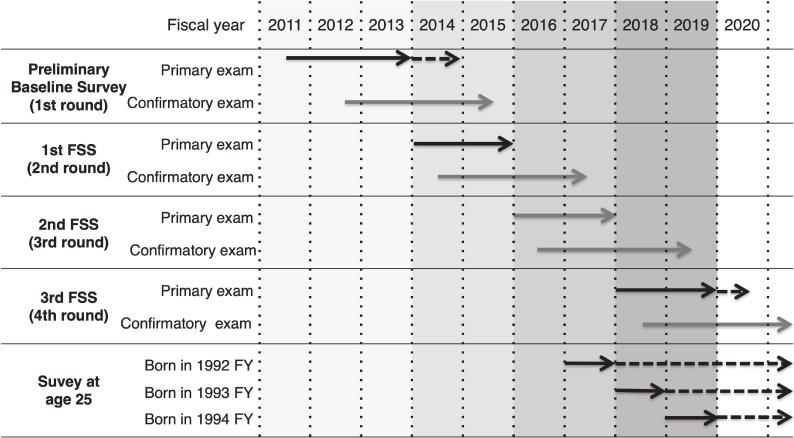
Progress of the Thyroid Ultrasound Examination program. FSS, Full-scale Survey.

The first-round survey, the Preliminary Baseline Survey, started on October 9, 2011, approximately 7 months after the accident, and most of the surveys were conducted until fiscal year (FY) 2013 (Figure 1).^8^ This survey had the characteristics of a prevalence survey and was conducted during the period when radiation-induced thyroid cancer was not expected to occur based on the studies on the Chernobyl nuclear accident; the study was estimated to clarify the prevalence of baseline thyroid cancer in Fukushima Prefecture and to contribute to future analyses (Table 1). After the first-round survey, a Full-scale Survey (FSS) with the characteristics of an incidence survey has been conducted at 2-year intervals. The 1st FSS, 2nd FSS, and 3rd FSS, which were second-, third-, and fourth-round surveys, were conducted from FY2014 to FY2015, FY2016 to FY2017, and FY2018 to FY2019, respectively (Figure 1). During this period, the results of the third-round survey have been concluded and the fifth-round survey is currently underway. Examination for subjects over 20 years of age are conducted every 5 years, and the survey for subjects aged 25 years has been conducted in parallel with the biennial examinations since FY2017 (Figure 1 and Table 1). This program was approved by the Ethics Review Committee of Fukushima Medical University (No. 1318). In the following sections, each survey is denoted by rounds of surveys beginning with the first-round survey.

**Table 1.** Structure of the TUE program

Round of survey	Survey category	Implementation period	Coverage
First round	Preliminary Baseline Survey (having the characteristics of a prevalence survey to clarify the prevalence of baseline thyroid cancer in the Fukushima Prefecture)	From October 2011 through March 2014	Residents who were born between April 2, 1992 and April 1, 2011
Second round	Full-scale Survey (conducted at 2-year intervals, with the characteristics of an incidence survey)	From April 2014 through March 2016	Residents who were born between April 2, 1992 and April 1, 2012
Third round	Full-scale Survey with Survey for residents aged 25 years (conducted with 5-years interval)	From May 2016 through March 2018	Residents who were born between April 2, 1994 and April 1, 2012 and born between April 2, 1992 and April 1, 1993
Fourth round	Full-scale Survey with Survey for residents aged 25 years	From April 2018 through March 2020	Residents who were born between April 2, 1996 and April 1, 2012 and born between April 2, 1993 and April 1, 1995

### Subjects

The subjects of the first-round survey consisted of 367,637 residents aged 0 to 18 years at the time of the accident and who were born between April 2, 1992 and April 1, 2011 (Table 1).^8^ Furthermore, in the FSS after April 2014, in addition to the subjects of the first-round survey, Fukushima residents born between April 2, 2011, and April 1, 2012, were targeted. In the second-round survey conducted between the FY2014 and FY2015, 381,244 people were targeted. The third-round survey targeted 336,670 people born between April 2, 1994 and April 1, 2012. The survey for residents aged 25 years was conducted with 22,653 Fukushima residents born between April 2, 1992, and April 1, 1993, in the third-round survey. The fourth-round survey targeted 294,240 people born between April 2, 1996, and April 1, 2012, with the survey of residents aged 25 years born from April 2, 1993, to April 1, 1995.

### Primary examination

**Figure 2.**
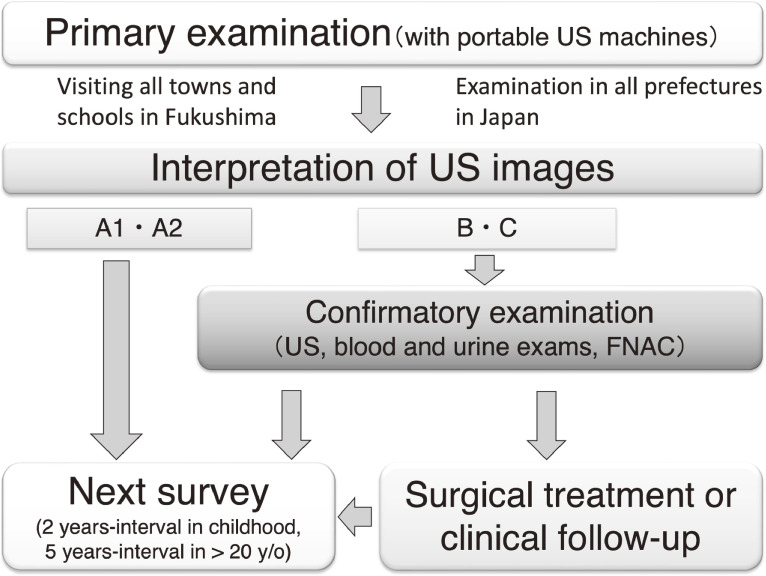
Flow chart of Thyroid Ultrasound Examination program

The TUE program consisted of primary and confirmatory examinations (Figure 2).^4^^,^^8^ First, the invitation letter for the primary examination was mailed to the subject’s home, even for examinations in schools. After obtaining written consent for the TUE by mail, the appointment for the primary examination was arranged. Primary examinations were performed mainly at schools, public facilities, and medical institutions in each area of Fukushima Prefecture and specialized medical institutions outside the prefecture by physicians and clinical and radiological technologists who were qualified to perform a thyroid examination. Interpretations of the primary examination were categorized as Grade A (A1, A2), B, or C (Table 2). Grade A referred to ultrasonographic findings considered to be within normal. Grade B referred to a nodule measuring ≥5.1 mm and/or a cyst measuring ≥20.1 mm in diameter. Grade C referred to a large suspicious thyroid nodule, overt extrathyroid extension, or large metastatic lymph node, which required immediate examination. Participants who were categorized with nodules of Grade B or C were encouraged to have a confirmation examination.

**Table 2.** Diagnostic criteria for thyroid nodules and cysts

Grade	Interpretation	Recommendation
A	Within normal	
(A1)	No nodules or cysts^a^	Next primary examination
(A2)	Nodules ≤5.0 mm^b^ and/or cysts ≤20.0 mm	Next primary examination
B	Nodules ≥5.1 mm and/or cysts ≥20.1 mm	Secondary examination
C	Required immediately examination	Urgent secondary examination

^a^Mixed cystic-solid nodule is included in the category of “nodules”.

^b^Nodules within the A2 category, which have ultrasonographic findings suggesting thyroid cancer with aggressive clinical features, may be classified as B.

### Informed consent in the primary examination

Until 2019, examinees and their families were informed of the disadvantages and objectives of the survey prior to the conduct of each primary examination, and examinations were conducted after obtaining an informed consent. During the Prefectural Oversight Committee Meetings in 2019, more detailed explanations were provided. Hence, the notification “Information on Thyroid Ultrasound Examination” was revised in 2020, with a special focus on the advantages and disadvantages of the examination. Easy-to-understand leaflets were also created for elementary and junior high school students. The advantages and disadvantages of this revised information were as follows:

1. Advantages of the examination:
  (1) If no abnormality was found through the examination, concerns related to the effects of radiation on health may be relieved, and the quality of life may be enhanced accordingly.
  (2) Early diagnosis and treatment may reduce the risk of surgical complications, risk of side effects due to treatment, and risk of recurrence.
  (3) Information concerning the existence or nonexistence of radiation effects can be provided through the analyses of examination results not only to the examinees and their families but also broadly to people in and outside of Fukushima Prefecture.
2. Disadvantages of the examination:
  (1) Diagnosis may lead to the treatment of cancers that otherwise would not show any symptoms or affect the quality and duration of life; diagnosing such cancers is considered overdiagnosis.
  (2) If a cancer or a suspected cancer is diagnosed early, the treatment and follow-up period can be prolonged and may increase psychological burdens or cause social and economic disadvantages.
  (3) Nodules or cysts that do not require treatment may be identified, and confirmatory examination or cytology may be recommended even in cases of benign nodules. This may impose physical burden and anxiety on the examinees and their families.

We also informed the examinees about the countermeasures for the abovementioned disadvantages:

For Disadvantage (1): Comprehensive measures were taken to avoid the diagnoses of nodules that do not require treatment by excluding nodules measuring ≤5.0 mm in diameter during the confirmatory examination and to determine the need for FNAC in patients with nodules of ≥5.1 mm by considering the imaging findings in line with the JABTS guidelines.

For Disadvantage (2): As a support program for the Fukushima Health Management Survey-Thyroid Examination, the Fukushima Prefecture government offers financial support to cover the medical costs necessary for the treatment and follow-up after the TUE.

For Disadvantages (2) and (3): The Fukushima Medical University has established the Mental Care Support Team, and its specialized staff help manage the anxieties of those who have been subjected to confirmatory examination. Additionally, Fukushima Medical University offers telephone consultation services in order to address any medical questions concerning the TUE results and thyroid disorders, to help manage psychological distress, and to hold explanation sessions at schools and other places.

### Confirmatory examination

If a B or C judgment was made in the primary examination, an invitation letter for a confirmatory examination was mailed to the participant. The confirmatory examination was performed after the written consent for it was obtained. The confirmatory examination included a medical examination, ultrasonographic examination using precision equipment, and blood/urine examinations. The blood examinations measured TSH, free T3, free T4, thyroglobulin, anti-thyroperoxidase antibody, anti-thyroglobulin antibody, and urinalysis measured urinary iodine concentration. FNAC was considered in accordance with the Japanese guideline for the implementation of FNAC (eMaterial 1).^7^^,^^9^ When FNAC was determined to be necessary, the purpose of FNAC, evidence of thyroid ultrasound findings indicating the necessity of FNAC, the method of FNAC, and possible complications of FNAC were explained to the examinees and their family. FNAC was performed only when consent was obtained.^10^ A repetitive FNAC for participants who had FNAC on a previous thyroid examination and had similar ultrasound results to their previous findings was not recommended.

The result of the confirmatory examination was explained directly to the examinee and his/her family, and after consultation, the medical plan that would be followed after the confirmatory examination was decided. When the result of the confirmatory examination was equivalent to A1 or A2 judgment in the judgment criteria at the time of the primary examination, in principle, the current round of examination was completed. In other cases, taking into account the examination results and the needs of the examinee and his/her family, the examinee was encouraged to undergo a medical follow-up or treatment or to undergo the next round of examination (Figure 2).

### Support for examinees and their families

Because TUE is not a mandatory examination and the examination is performed with informed consent, it is important to ensure that the subjects and their families have an understanding of thyroid examinations. To keep them updated about this program, the TUE newsletter was issued and mailed to all the patients that were to be examined and their parents twice annually. This consisted of the examination plan, results, explanations of topics, and a question-and-answer section. In addition, upon request, we held on-site lessons or dialogues with schools and local communities. Using programs and materials tailored to each age group, we explained what the participants needed to know about the thyroid gland and ultrasonography, such as the purpose of TUE, the advantages and disadvantages of the examination, thyroid cysts, nodules, and cancer.

To support the participants before the primary examination, a booklet titled “Examination Notice” was mailed to explain the purpose of the examination and its advantages and disadvantages. In addition, to provide sufficient information for the decision making of agreement or disagreement for the examination, the participants and their families were supported with the call center, medical hot lines, and web consultations.

In the primary examinations at general venues in Fukushima Prefecture, a tentative explanation of the results of the primary examinations by medical doctors was presented using the ultrasound images. At the other venues, leaflets with explanations of thyroid cysts and nodules and confirmatory examinations were distributed to increase their understanding of the examination results.

To measure the worries and anxieties caused by the examinations of those who were participants in the confirmatory examination, a support team consisting of clinical psychologists, medical social workers, and nurses commenced mental care support. The support team provided psychological support by creating a close bond with the examinees and their families, listening closely to their anxieties and questions, and providing support so that they could talk to the doctor effectively. The activities of the support team were not only available at the time of the confirmatory examination, but also after the transition to medical follow-up or treatment.

## RESULTS

### Results of the TUE program

**Figure 3.**
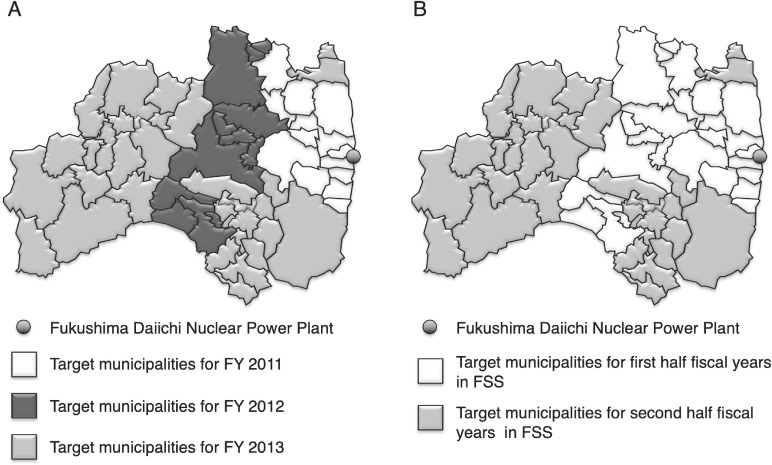
Target municipalities for the first-round survey (**A**) and Full-scale Survey (**B**)

The Preliminary Baseline Survey (first round) and FSSs (second round and beyond) were conducted according to the level of ambient radiation dose (Figure 3). Table 3 shows the results of the first-round survey,^11^ the surveys on second to fourth rounds,^12^^–^^14^ and the survey at age 25 years.^15^ The participation rate of the primary examination in the first-, second- and third-round surveys were 81.7%, 71.0%, and 64.7%, respectively. The rates of Grade B in the primary examination were 0.8%, 0.8%, and 0.7% in the first-, second-, and third-round surveys, respectively. As of June 30, 2021, 116, 71, 31, and 36 examinees in the survey of first, second, third, and fourth rounds of survey, respectively, and 9 examinees in the survey for those aged 25 years were determined to have nodules cytologically diagnosed as malignant or suspicious for malignancy (hereafter referred to as cytologically malignant nodules). The confirmatory examination of the fourth-round survey and the primary and confirmatory examination of the fifth-round survey are currently in progress.

**Table 3.** Results of Thyroid Ultrasound Examination

			First round^a^	Second round^b^	Third round^c^	Fourth round^d^	Survey at age 25 years^e^
Fiscal year^f^			2011–2013	2014–2015	2016–2017	2018–2019	2017–
Subjects			367,637	381,237	336,667	294,237	87,694
*The primary examination*							
	Participants for the primary exam		300.472	270,552	217,922	183,352	7,621
	Participation rate		81.7%	71.0%	64.7%	62.3%	8.7%
	Examination result confirmed		300.472	270,552	217,922	183,338	7,260
	Grade in the primary examination	A1	51.5%	40.2%	35.1%	33.6%	42.7%
		A2	47.8%	59.0%	64.2%	65.6%	52.3%
		B	0.8%	0.8%	0.7%	0.8%	4.9%
		C	0.0%	0.0%	0.0%	0.0%	0.0%
*The secondary examination*							
	Subjects for the secondary exam		2,293	2,230	1,502	1,391	359
	Participants		2,130	1,877	1,104	1,021	239
	Participation rate		92.9%	84.2%	73.5%	73.4%	66.6
	Examination result confirmed		2,091	1,834	1,068	991	227
	Equivalent to B judgment		1,380	1,404	959	898	210
	FNAC performed		547	207	79	87	17
	Malignant or suspicious (FNAC)		116	71	31	36	9
*Results in clinical practice*							
	Examinees surgically treated		102	52	29	29	6
	Pathological diagnosis	PTC	100	51	29	29	5
		FTC					1
		PDTC	1				
		Others (cancer)		1			
		Benign	1				

FNAC, fine needle aspiration cytology; FTC, follicular thyroid carcinoma; PDTC, poorly differentiated thyroid carcinoma; PTC, papillary thyroid carcinoma.

^a^Data were from the reports of Preliminary Baseline Survey as of March 31, 2017.^10^

^b^Data were from the reports of the Full-scale Survey (second round survey) as of March 31, 2021.^11^

^c^Data were from the reports of the Full-scale Survey (third round survey) as of March 31, 2021.^12^

^d^Data were from the reports of the Full-scale Survey (fourth round survey) as of June 30, 2021.^13^

^e^Data were from the reports of Survey at age 25 years as of March 31, 2021.^14^

^f^Fiscal and academic year in Japan begins on April 1 of a given calendar year and ends on March 31 of the next calendar year.

The fourth-round survey and survey at age 25 years are currently in progress, and the corresponding results show the results of the progress.

### Analytical results of the TUE

#### Detection of nodules cytologically diagnoses as malignant or suspicious for malignancy

In Chernobyl, more cases of childhood thyroid cancer were found in the younger age group at the time of the accident.^16^ In the first-round survey, most of the cases with cytologically malignant nodules were at least 10 years old at the confirmatory examination,^11^ and the number of diagnosed cases tended to increase in proportion with age (Table 4).^11^ In a study of participants who had undergone the first-round survey until the FY2013, the adjusted detection rate corrected by the implementation rate of confirmatory examinations showed an age-dependent increase in the detection rate and was observed to be higher in female participants than in male participants.^10^ When the maximum diameter of cytologically malignant nodules was classified into 5.1 to 10.0 mm, 10.1 to 20.0 mm, and 20.1 mm or more, malignant nodules within 10.1 to 20.0 mm were predominant.^10^

**Table 4.** Detection rate of cases diagnosed as malignant or suspicious for malignancy in the first-round, the second-round, and the third-round survey

Age, years^a^	First round			Second round			Third round^b^		

	Participant^c^	Malignant^d^		Participant^c^	Malignant^d^		Participant^c^	Malignant^d^	

	(*n*)	(*n*)	(%)	(*n*)	(*n*)	(%)	(*n*)	(*n*)	(%)
0–4	40,663	0	0.000	19,436	0	0.000	1,326	0	0.000
5–9	84,156	1	0.001	75,121	2	0.003	61,375	0	0.000
10–14	95,999	21	0.022	89,197	17	0.019	81,666	10	0.012
15–19	70,510	77	0.109	70,560	36	0.051	63,581	18	0.028
20–24	9,137	17	0.186	16,238	16	0.099	10,870	4	0.037
≥25	0	—	—	2	0	0	1,356	2	0.147
Total	300,465	116	0.039	270,554	71	0.026	220,174	34	0.015

All date were as of March 31, 2020 and from the reports in the materials of 16th Subcommittee for TUE program.^17^

^a^Age at the primary examination.

^b^The result of the third-round survey included the results from the survey at age 25 years for subjects born in the 1992 fiscal year.

^c^Number of participants for the primary examination.

^d^Number and detection rate of cases with nodules cytologically diagnosed as malignant or suspicious for malignancy.

In the second-round survey and the third-round survey, which included examinations performed at the same time for participants aged 25 years, the number of cases with cytologically malignant nodules is also shown in Table 4.^17^ The increase in the number of cases with malignant nodules in proportion to the age and the minimum age for detection in second- and third-round surveys, which were characterized as incidence surveys, was similar to that in the first-round survey, which was characterized as a prevalence survey.

**Figure 4.**
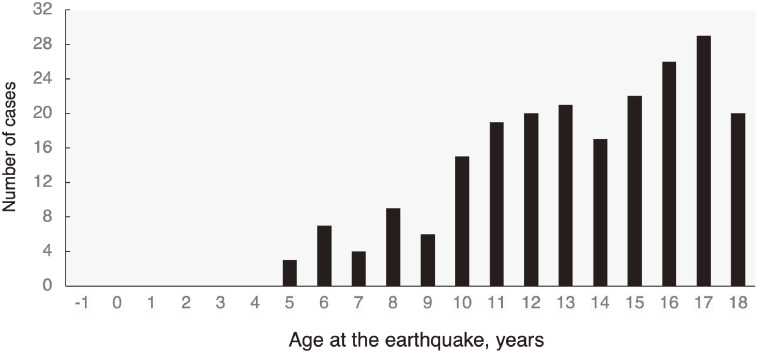
Total number of cases diagnosed as malignant or suspicious for malignancy in the first-round, the second-round, and the third-round survey for each age at the time of the earthquake

The total number of malignant cases according to the age at the time of the earthquake from the first to the third-round survey is shown in Figure 4. Up to the third-round survey, the youngest cases with cytologically malignant nodules were aged 5 years at the time of the earthquake, and the number of detected cases increased in proportion to the age at the time of the earthquake.^12^^,^^13^ In particular, thyroid cancer was not detected in the first-round survey among examinees who were ≤5 years old at the time of the disaster.

The detection rate of cytologically malignant nodules among all participants was 0.039% for the first-round survey, 0.026% for the second-round survey, and 0.015% for the third-round survey, with a decreasing trend as the number of examinations increased (Table 4).^18^ Age-dependent increases in the detection rate of cytologically malignant nodules were observed in all rounds of surveys.^18^ The incidence rate of cytologically diagnosed thyroid cancer in the second-round survey in each age group was calculated from the intervals between the two examinations for participants who had undergone both the first- and second-round surveys.^19^ This study also showed an increase in the incidence rate of cytologically malignant nodules in parallel with the age at examination.

#### Relationship of the level of radiation dose and regions in Fukushima with the detection rate of malignant cases

In the first-round survey, the prevalence of cytologically malignant nodules was 37.3 out of 100,000 in whole area in Fukushima, and the estimated the level of external radiation dose for 4 months according to the Basic Survey in the Fukushima Health Management Survey for all residents in Fukushima was below 2.2 mSv.^20^

In the regional analysis of the detection rate of cytologically malignant nodules in the first-round survey, the presence or absence of general geographical variability of the thyroid cancer and the sex- and age-standardized prevalence in Fukushima Prefecture was examined using flexibly shaped spatial scan statistics, maximum excess events tests, Poisson regressions, and simulation-based sensitivity tests.^21^ This study found no significant spatial anomalies/clusters or geographic trends of thyroid cancer prevalence among the ultrasound examinees, indicating that the thyroid cancer cases detected were unlikely to be attributable to regional factors.

Based on the results of the individual external exposure dose in the Basic Survey, the municipalities in Fukushima Prefecture were classified into three groups according to the external exposure dose (Group A for the area where many people were presumed to have had a relatively high dose; Group B for the area with the middle dose; and Group C for the area with the lowest dose). In the first-round survey, the sex- and age-adjusted odds ratios (ORs) for the risk of thyroid cancers with reference to Group C were 1.49 (95% confidence interval [CI], 0.36–6.23) in Group A and 1.00 (95% CI, 0.67–1.50) in Group B.^22^

Furthermore, based on the results of the Basic Survey, the municipalities in Fukushima Prefecture were reclassified into five groups according to their external exposure dose and their relationship with the detection rate of cytologically malignant nodules found 4 years after the earthquake was determined. In this study, no association was found between the thyroid cancer detection rate and regional differences in the external exposure dose.^23^ These studies mentioned in this section indicated that there were no regional differences in the detection rates of thyroid cancer in first-round survey.

The analysis of the results obtained from the second-round survey for only those who also had undergone first-round survey, no relationship was found between the external exposure dose and the detection rate of cytologically malignant nodules.^24^

The thyroid gland is an organ that accumulates iodine, and in a nuclear power plant accident, internal exposure due to the uptake and accumulation of radioactive iodine is one of the major factors in the development of thyroid cancer. Therefore, the relationship between the detection rate of cytologically malignant nodules and the estimated thyroid absorbed dose of individuals from the municipalities in Fukushima, which was the sum of the external and internal exposure doses announced by the United Nations Scientific Committee on the Effects of Atomic Radiation (UNSCEAR) in 2013,^25^ was analyzed. In the first- and the second-round survey, an increase in the detection rate in proportion to the exposure dose was not observed.^26^ Based on these findings, UNSCEAR suggested in the 2020 report that the high thyroid cancer risk that could be inferred from exposure to radiation exposure was most likely not discernible in any of the age groups considered.^27^

#### Factors related to the detection and enlargement of thyroid cancer

In order to identify the factors related to the enlargement of thyroid cancer, related parameters associated with the changes in the diameter of thyroid cancer between the primary and confirmatory examinations were analyzed by dividing the three groups based on the changes in the tumor diameter: by increasing, shrinking, or no change.^28^ No significant differences were observed in age, sex, tumor diameter, observation period between primary and confirmatory examinations, or parameters in blood examinations among the three groups during an average 6-month period. Although there was no significant correlation between the period and the logarithmic index of volume change (Pearson *R* = 0.121; 95% CI, −0.062 to 0.297), the coefficient of growth (year^−1^) showed a significant and negative correlation with the tumor diameter that did not show a normal distribution (Spearman ρ = −0.183; 95% CI, −0.354 to −0.001), suggesting a reduction in the growth rate with time.^28^ In addition, body mass index^24^ and the level of thyroid stimulating hormone^29^ have been found to be associated with the detection of thyroid cancer in the first-round survey.

#### Detection rate of thyroid nodules

For the participants who participated in the examination within 3 years after the earthquake, the detection rate of thyroid nodules was analyzed in the first-round survey.^10^ Thyroid nodules were found in 1.0% and 1.7% of male and female examinees, respectively, with a significantly higher rate being observed in females. In addition, an age-dependent upward trend in the detection rate was observed in females aged ≥10 years and males aged ≥14 years, and the sex difference was evident in those aged ≥10 years. Of the patients with nodules, 13.0% of males and 15.0% of females had multiple nodules. Also, when the maximum diameter of the nodule was divided into 5.0 mm or smaller, 5.1 to 10.0 mm, 10.1 to 20.0 mm, and 20.1 mm or larger, the detection rate of 5.0 mm or smaller was the most frequent among those under 10 years old, while the detection rate of nodules of 5.1 to 10.0 mm was the highest in examinees ≥10 years old. An age-dependent increase in the detection rate was observed in all groups.

#### Detection rate of thyroid cysts

Among the examinees who participated in the first-round survey, the thyroid cyst detection rate was 45.7% for males and 50.0% for females while the detection rate in females tended to be slightly higher.^10^ An age-dependent increasing trend was observed up to the age of 10 years, peaking in the age range of 11–12 years. While there was a decreasing tendency observed at 13 years and older, it was more noticeable in the detection rate of cysts measuring 3 mm or smaller. However, the detection rate of cysts ≥5.1 mm showed an increasing tendency even after 13 years of age. The median maximum diameter of cysts increased in proportion with the age. In addition, the proportions of multiple cysts found in males and females were 89.3% and 89.6%, respectively.

#### Other findings in TUE

Other findings in addition to cysts and nodules have been obtained from the TUE. One of which is the ectopic thymus of the thyroid gland, for which caution is required because the ultrasound image can resemble thyroid cancer. An ectopic thymus in the thyroid gland was observed in 375 (0.99%) of 37,816 participants.^30^ The detection rate of an intrathyroidal ectopic thymus showed an inverse relationship with age. In addition, the three-dimensional sizes of both lobes of the thyroid gland and a rare congenital variant characterized by the lack of thyroid lobe development were determined.^31^^,^^32^

#### Analysis of characteristics of non-examinees in the TUE program

The relationships between the proportion of non-examinees in the first-round survey and the characteristics of individuals living in Fukushima Prefecture on March 11, 2011, were analyzed.^33^ In particular, the differences in the odds of non-examinees between residential areas and the influence of changing residences (moving) after the Great East Japan Earthquake were determined. The logistic regression analyses of the dataset included 64,117 primary examination non-examinees and 194 confirmatory examination non-examinees. This indicated that females were more likely to participate in the primary examination compared to males, with an adjusted OR of 0.80 (95% CI, 0.78–0.81) for the proportion of non-examinees adjusted for age, categorized area of residence on March 11, 2011 based on exposure to radiation, and moving after the Great East Japan Earthquake. The OR for the proportion of non-examinees in the primary examination was the lowest among children aged 6–10 years (OR 0.26; 95% CI, 0.25–0.27), while it was the highest among those >16 years old (OR 5.30; 95% CI, 5.16–5.43) compared with children aged 0–5 years. Individuals residing in the western part of the prefecture had higher ORs (ie, less likely to participate in the primary examination). The proportion of non-examinees was higher among those who moved from the municipalities residing before March 11, 2011 to the current address after the accident than among those who did not (OR 1.72; 95% CI, 1.64–1.79). These results were considered to be due to the fact that the subjects who moved out of the prefecture and those who graduated from high school were unable to participate this examination at schools, but only at general venues such as public facilities or private halls or in authorized medical institutions that varied regionally in number. Hence, it was considered necessary to increase the number of medical institutions and provide different opportunities to undergo TUE in the areas of residence where the target persons are actually residing, including those living outside of Fukushima Prefecture.

## DISCUSSION

### Evaluation of the TUE results from the committees of Fukushima Prefecture

The Prefectural Oversight Committee, consisting of outside experts, was set up to evaluate the Fukushima Health Management Survey, including the TUE program, and to obtain advice from a wide range of professional perspectives. To evaluate the results of the TUE in detail, the Subcommittee for TUE was established in 2013. Based on the discussion in the Subcommittee for the TUE program, the Prefectural Oversight Committee released the comments on the first-round survey^34^ and the second-round survey.^35^

For the first-round survey, the committee made the following evaluations in March 2016.^34^ The detection of thyroid cancer was evaluated to be unlikely affected by radiation for the following reasons. The exposure dose was generally lower than that in the Chernobyl accident, and the period from exposure to cancer detection was as short as 1 to 4 years. Additionally, thyroid cancer was not detected in examinees aged 5 years or younger at the time of the accident. Furthermore, there was no significant difference in the detection rate between regions in Fukushima Prefecture, where they lived at the time of the accident. Although the influence of the radiation exposure was estimated to be low, it could not be completely ruled out at this time. Therefore, the committee recommended that while a long-term examination would be indispensable for the assessment of the impact of radiation exposure, it should be accompanied by a careful explanation of the disadvantages of this examination. This report also mentioned that thyroid cancers were found to be at a rate of times higher in comparison with prevalence of thyroid cancer estimated by the population-based cancer registry in Japan.^34^ In this regard, the possibility of overdiagnosis (ie, diagnosis of cancer that does not threaten life expectancy or cause symptoms) was noted, although the possibility of excess incidence due to exposure cannot be completely denied. However, some of committee members have pointed out that, even in the cases that are assumed to be overdiagnosis at present, many might be detected as cancers that threaten life expectancy or cause symptoms not only within a few years but also later.

The committee made the following evaluations in July 2019 for the second-round survey.^35^ At this point, no relationship between thyroid cancer and radiation exposure was found in the second-round survey. The reason for this was that no dose-effect relationship was observed in Fukushima based on the level of absorbed radiation dose in thyroid gland estimated by UNSCEAR.^25^^,^^26^ In addition, the detection rate of cytologically malignant nodules increased with increasing age at the time of the accident was different from that of the cases after the Chernobyl accident.

### Comparison of the data between residents in Fukushima and those out of Fukushima

The first-round survey showed that cysts and nodules were found in approximately half and 1.3% of examinees, respectively. Since the prevalence of thyroid nodules and cysts in Japanese children has not been clarified,^36^ children in three prefectures (Aomori, Yamanashi, and Nagasaki Prefectures) were examined to investigate the frequency of thyroid nodular lesions in the general population.^37^ These examinations were performed using the same ultrasound procedures as those used in the Fukushima Health Management Survey. The number of participants living in three prefectures that were analyzed was 4,365, and the ages ranged from 3 to 18 years old. Thyroid ultrasonography revealed that 42.5%, 56.5%, 1.0%, and 0.0% of participants received a judgment of Al, A2, B, and C, respectively.^37^ In addition, cysts were found in 56.9% of the participants, and nodules in 1.7%. These results were similar to those of the first-round survey.^37^^,^^38^

Since further examination is required to clarify the clinical diagnosis of these nodules detected in the children residing in these prefectures, a follow-up study was conducted.^39^ Of the 44 participants who were judged as B in the initial study in three prefectures, 31 provided consent for the follow-up study and cooperated in the investigation of thyroid nodules. As a result, 11 (35%) and 20 examinees (65%) were diagnosed as equivalent to A and B, respectively. Most cases were diagnosed with cysts and benign nodules; FNAC was performed in two, and one was diagnosed with thyroid cancer.

Few reports studying the prevalence of thyroid cancer in children and adolescents are available.^36^ Katanoda et al reported an estimated prevalence of 0.002% among those under 20 years of age based on the 2014 thyroid cancer incidence rate,^40^ suggesting that the prevalence of thyroid cancers found in this examination was higher than that estimated by national cancer registries. However, in the survey in three prefectures mentioned above, detection rate of thyroid cancer was 1/4,365 with a participation rate of 31/44 for the follow-up study. Screening of thyroid diseases by palpation discovered thyroid cancer in 2 of 5,179 school children (0.04%) in Utah, Nevada, and Arizona.^41^ In Japan, 9,988 students aged over 18 years at Chiba University underwent thyroid screening with palpation, and 4 students (0.03%) were diagnosed with thyroid cancer.^42^ These studies using palpation and ultrasonography have reported higher detection rates of thyroid cancer, similar to the detection rate of cytologically malignant nodules in TUE in Fukushima.

### Efforts for the risk of overdiagnosis in the TUE program

In adults, the detection rate of nodules in the thyroid gland using thyroid ultrasonography was high at 19–35%^36^; most of these were benign adenomatous nodules formed by hyperplasia. However, the detection rate of thyroid cancer was approximately 0.5%, with papillary thyroid carcinoma accounting for the most.^36^ In addition, it is known that latent cancers are found in the thyroid gland in about 10% of cases at autopsy, most of which are micropapillary cancers with a diameter of smaller than 5 mm.^43^

The fact that low-risk thyroid cancers are frequently detected indicates that thyroid cancer is one of the malignant tumors with a risk of overdiagnosis, which is defined as the diagnosis of a disease that does not cause death or symptoms over a lifetime.^44^ In this context, if a patient is already symptomatic at the time of detection, there is almost no possibility of overdiagnosis. However, in asymptomatic cases, thyroid cancer could theoretically be overdiagnosed, regardless of whether standard treatment is required. In situations where clinical evidence indicates the need for standard treatment, diagnosis and surgical treatment are performed as the disadvantages of worsening cancers due to a postponed diagnosis are considered significant.^45^ On the contrary, if standard treatment is not recommended, the risk of overdiagnosis is high; hence, it is preferable to postpone the diagnosis and the subsequent follow-up, and to diagnose and treat the patient only when cancer progression is observed. Therefore, in Japan, the risk of overdiagnosis is reduced by limiting the diagnosis to cases in which diagnosis and treatment are required.

Compared to the guidelines of other countries, measures against the risk of overdiagnosis are implemented earlier in Japan (eMaterial 1). Guidelines have been published to establish criteria for FNAC^9^^,^^46^^–^^48^ and to recommend the minimum necessary surgical treatment.^45^^,^^49^^,^^50^ In recent years, discussions have been held with other countries with regard to the medical treatment of low-risk papillary thyroid carcinoma, and more careful diagnoses and treatment have been recommended in recently published guidelines.^51^^–^^55^

Thyroid cancer in children and adolescents is generally considered to have a good prognosis; however, compared to adults, there is less knowledge about the natural history of cancer, and more careful treatment is required. Nevertheless, following the guidelines of relevant academic societies, the TUE program has taken the following steps to reduce the risk of overdiagnosis. In the primary examination, nodules of 5.0 mm or smaller were judged as A2, and the confirmatory examination was not recommended. This measure would eliminate most latent cancers.^4^^,^^56^ In the confirmatory examination, the indication for FNAC was evaluated according to the guidelines for the implementation of FNACs published by the Japanese Society of Breast and Thyroid Sonology.^7^^,^^56^ These measures can reduce excessive FNACs and postpone the diagnosis of low-risk thyroid cancer. In fact, we recently reported that 0.6% and 0.4% of nodules measuring ≤5.0 mm and 5.1–10.0 mm found in the first-round survey are diagnosed as malignant or suspicious for malignancy in the second-round survey.^57^

Even in the TUE program that implemented these efforts against overdiagnosis, the detection rate of thyroid cancer was more than 10 times higher in the TUE program than the estimated thyroid cancer incidence rate in the national cancer registry, as mentioned above. The following study have suggested that these high detection rates might be a reflection of the early diagnosis of cancers by ultrasonography.^58^ The detection rate of cytologically malignant nodules in the first-round survey was evaluated from a model constructed using the cancer registration data of the National Cancer Center on the assumption that there was no effect from radiation and the sensitivity of each step of this examination. This study showed that the actual number of detections was within the range of the 95% CI of the number of detections predicted from the model.^58^

### Feedback from the results of surgical treatment

In the Prefectural Oversight Committee for Fukushima Health Management Survey, 101 cases from the first-round survey, 52 cases from the second-round survey, 29 cases from the third-round survey, 29 cases from the fourth-round survey, and 6 cases from the survey at the age of 25 years have been reported as cases pathologically diagnosed as thyroid cancer (Table 3).^11^^–^^15^ Analysis of these surgically treated cases provided important feedback regarding TUE.

#### Pathological diagnosis of surgical cases

The pathological diagnosis was papillary thyroid carcinoma in 214 cases, follicular cancer in one case, poorly differentiated cancer in one case, and other thyroid cancer in one case (Table 3).^3^^,^^59^ In addition, as of the end of July 2017, 11 cases of thyroid cancer were not reported because they were out of the scope of the Fukushima Prefecture Health Survey; however, they underwent surgery at Fukushima Medical University Hospital. These cases corresponded to 5.7% of 194 cases with cytologically malignant nodules in the TUE program as of the end of July 2017.^60^

#### Clinical characteristics of surgical cases

Details of the surgical cases were reported for 125 patients who had undergone surgery at Fukushima Medical University Hospital on March 31, 2016.^61^ The sex ratio (male:female) was 1:1.8 and was higher in females; the average tumor diameter immediately before surgery was 14 mm (5.0 to 53 mm); 121 (96.8%) patients had lesions within a lobe, and four (3.2%) had lesions in both lobes. Comparing the postoperative findings, preoperative EX1 (extrathyroidal infiltrations to sternohyoid muscle or surrounding adipose tissue) was 9.6%, while the postoperative EX1 was 39.2%. Preoperative lymph node metastasis was observed in 22.4% of patients, while postoperative lymph node metastasis was observed in 77.6%. All three M1 (with distant metastasis) cases were suspected of having lung metastases. Bilateral lesions were observed in only three cases. Total thyroidectomy was performed in only 11 cases (8.8%), and lobectomy in 114 cases (91.2%).

In Chernobyl, 68% of surgically treated cases underwent total or subtotal thyroidectomy, and these were subsequently treated with radioactive iodine.^62^ However, in Fukushima, more than 90% underwent lobectomies, and radioiodine treatment was rarely performed. These conservative treatments, an approach to treating thyroid cancers utilizing limited range of surgical procedure rather than total thyroidectomy combined with radioiodine therapy, have resulted in the development of rare postoperative complications in patients in Fukushima Prefecture.^3^

#### Gene mutations in thyroid cancer

In Chernobyl, especially in patients with thyroid cancer with a short latent period of 7–10 years, 64–86% of them had *RET/PTC* chromosomal rearrangement mutations, especially *RET/PTC3* rearrangements.^63^ Analyses of driver mutations in 67 papillary thyroid carcinomas and one poorly differentiated thyroid carcinoma in Fukushima detected BRAFV600E in 43 cases (63.2%), *RET/PTC1* in six (8.8%), *RET/PTC3* in one (1.5%), and *ETV6/NTRK3* in four (5.9%). The genetic pattern was completely different from that of the post-Chernobyl papillary thyroid carcinomas. Furthermore, in a study of 138 cases including additional cases, the BRAF V600E point mutation was observed in 69.6% of cases,^64^ suggesting that it was different from the gene mutation in the thyroid cancer that occurred in Chernobyl. The profile of the genetic alterations is useful for understanding the characteristics of cancer. *RET/PTC3* rearrangement has been found in pediatric post-Chernobyl thyroid cancer with a latency of 5–10 years.^63^^,^^65^ In these cases, the solid variant of papillary thyroid carcinoma had a strong correlation with *RET/PTC3*.^66^ On the contrary, *RET/PTC3* rearrangement with papillary carcinoma was detected in only one patient in the study conducted in Fukushima. These data indicate that the cancer characteristics in the Fukushima cases were different from those in post-Chernobyl papillary cancer cases. However, previous studies reported that the frequency of *RET/PTC3* rearrangement is higher in areas with lower iodine intake, regardless of the degree of radiation exposure, and the relationship between *RET-PTC3* and radiation remains unclear.^66^

It has also been clarified that cases with a BRAFV600E mutation tend to have smaller tumor size and a higher rate of lymph node metastasis than cases without a BRAFV600E mutation.^64^

#### Efforts to address the risk of overtreatment in medical care after the TUE

Patients diagnosed as malignant or suspicious for malignancy using cytological examination in the TUE were referred to the medical department. However, in Japan, active surveillance is conducted in adult patients with very low-risk papillary thyroid carcinoma (T1aN0M0) with no evidence of metastasis or extension, under an appropriate medical care system after obtaining the patient’s consent, following an adequate explanation of the disease condition and the risks/benefits of the management.^45^^,^^50^

Analysis was conducted for clinical characteristics of cases with very low-risk papillary thyroid carcinomas that may be subject to active surveillance in patients referred to the surgical department.^61^ This analysis identified 44 cases of T1aN0M0 (tumor diameters of 1 cm or smaller, no extrathyroidal infiltrations, or lymph node/distant metastases) in the preoperative evaluation. Thirty-three patients underwent surgery due to the following reasons (with duplication): 20 with suspected EX1 (extrathyroid infiltration = classified as T3), 3 with suspected lymph node metastases, 10 with suspected recurrent laryngeal nerve infiltrations, seven with suspected tracheal infiltrations, one with Graves’ disease, and one with lung shadow. In addition, 11 patients were treated surgically according to the wishes of these patients and their families even after receiving recommendations for non-surgical follow-ups. Of the above 44 patients, only 5 had T1aN0M0, and 39 (89%) had lymph node metastasis or extrathyroidal infiltrations as the postoperative pathological diagnosis. These results were based on the criteria for performing FNAC^7^ as part of the confirmatory examination of the TUE program, based on the diameter of the nodule and the findings on ultrasonography; for nodules ≤1 cm, FNAC was performed only when almost all findings were malignant. The diagnostic strategy (eMaterial 1) for thyroid nodules in the TUE program may contribute to the postponement of the diagnosis of thyroid cancers eligible for active surveillance until the next round of the survey.

### Lessons learned from the Chernobyl nuclear power plant accident

On April 26, 1986, the worst nuclear accident in human history occurred at the Chernobyl reactor #4 in the Ukrainian Republic of the former Soviet Union. The amount of ^131^I released from the reactor was about 10 times higher than that released during the Fukushima accident.^27^ Of the late effects of radiation in the surrounding population, the only proven health hazard was thyroid cancer in the young generation. Unlike Fukushima Prefecture, the area around the Chernobyl nuclear power plant was relatively iodine deficient. In addition, evacuation and disposal of raw milk and other foodstuffs were carried out in Fukushima Prefecture, while the residents in the area around the Chernobyl nuclear power plant were not informed of the accident and were exposed to the internal radiation through ingestion of milk and food contaminated with radioactive materials.

After the Chernobyl accident, an increase in the number of childhood thyroid cancers was reported based on the data from the cancer registry in Belarus.^67^^,^^68^ Subsequently, case-control studies^69^ and cohort studies^70^^,^^71^ conducted with the cooperation of international specialists revealed that the risk of thyroid cancer increased in people exposed to relatively high doses of thyroid radiation. In Chernobyl, an increase in the incidence of thyroid cancers was also found in residents under medical surveillance, including those who underwent ultrasound examinations,^3^ indicating that precise case-control and cohort studies associated with the level of radiation dose are important in assessing the radiation risk. From the lessons learned from the Chernobyl accident, the TUE program, with the characteristics of a cohort study, is being implemented to estimate the radiation dose absorbed in the thyroid and to conduct case-control studies correlating these doses. The final conclusions will await the results of the careful investigation.

### Conclusion

In the UNSCEAR report published in 2020,^27^ the absorbed thyroid dose was estimated to be lower than the dose estimated in the UNSCEAR 2013 Report.^25^ The results obtained from the TUE program did not indicate an increase in the incidence of childhood thyroid cancer due to radiation exposure in Fukushima Prefecture. The UNSCEAR 2020 report^27^ also concludes that a substantial number of pediatric thyroid cancers detected in Fukushima do not appear to be associated with radiation exposure, but rather a result of the performance of highly sensitive ultrasound screening procedures.

The TUE program has been implemented mainly as a health-supportive program for those who wish to be examined, rather than as a study to scientifically analyze the effects of radiation exposure in which all residents are required to participate. In addition, it is considered somewhat difficult to clarify the effects of exposure to radiation in Fukushima because the radiation dose is clearly lower than that of the Chernobyl nuclear power plant accident. However, in order to meet the expectations of the residents of Fukushima Prefecture, we will continue our efforts to reduce the residents’ anxiety related to the health hazards caused by radiation and to clarify the effects of radiation in Fukushima Prefecture by conducting accurate examinations and precise analyses.

## SUPPLEMENTARY MATERIAL

eMaterial 1. Supplemental methods

## References

[1] Takamura N, Yamashita S. Lessons from Chernobyl. Fukushima J Med Sci. 2011;57:81–85. 10.5387/fms.57.81 22353657

[2] Yasumura S, Hosoya M, Yamashita S, ; Fukushima Health Management Survey Group. Study protocol for the Fukushima Health Management Survey. J Epidemiol. 2012;22:375–383. 10.2188/jea.JE20120105 22955043 PMC3798631

[3] Yamashita S, Suzuki S, Suzuki S, Shimura H, Saenko V. Lessons from Fukushima: latest findings of thyroid cancer after the Fukushima nuclear power plant accident. Thyroid. 2018;28:11–22. 10.1089/thy.2017.0283 28954584 PMC5770131

[4] Yamashita S, Suzuki S. Risk of thyroid cancer after the Fukushima nuclear power plant accident. Respir Investig. 2013;51:128–133. 10.1016/j.resinv.2013.05.007 23978638

[5] Yasumura S, Ohira T, Ishikawa T, . Achievements and current status of the Fukushima Health Management Survey. J Epidemiol. 2022;32(Suppl 12):S3–S10. 10.2188/jea.JE20210390 PMC9703928 36464298

[6] Kitaoka M, Miyamoto Y, Fukunari N, . Ultrasound diagnostic criteria for thyroid nodule. Jpn J Med Ultrasonics. 2011;36:669–670.

[7] Suzuki S. Childhood and adolescent thyroid cancer in Fukushima after the Fukushima Daiichi Nuclear Power Plant accident: 5 Years On. Clin Oncol (R Coll Radiol). 2016;28:263–271. 10.1016/j.clon.2015.12.027 26822892

[8] Suzuki S, Yamashita S, Fukushima T, . The protocol and preliminary baseline survey results of the thyroid ultrasound examination in Fukushima [Rapid Communication]. Endocr J. 2016;63:315–321. 10.1507/endocrj.EJ15-0726 26924746

[9] Shimura H, Matsumoto Y, Murakami T, Fukunari N, Kitaoka M, Suzuki S. Diagnostic strategies for thyroid nodules based on ultrasonographic findings in Japan. Cancers (Basel). 2021;13(18):4629. 10.3390/cancers13184629 34572857 PMC8464767

[10] Shimura H, Sobue T, Takahashi H, ; Thyroid Examination Unit of the Radiation Medical Center for the Fukushima Health Management Survey Group. Findings of thyroid ultrasound examination within 3 years after the Fukushima Nuclear Power Plant accident: the Fukushima Health Management Survey. J Clin Endocrinol Metab. 2018;103:861–869. 10.1210/jc.2017-01603 29253182

[11] Fukushima Medical University. Supplemental Report of Preliminary Baseline Survey. 2017, http://kenko-kanri.jp/en/health-survey/document/pdf/27_5Jun2017.pdf.

[12] Fukushima Prefecture. Report of Full-scale Survey (Second Round Survey). 2021, https://www.pref.fukushima.lg.jp/uploaded/attachment/461564.pdf (in Japanese).

[13] Fukushima Prefecture. Report of Full-scale Survey (Third Round Survey). 2021, https://www.pref.fukushima.lg.jp/uploaded/attachment/461565.pdf (in Japanese).

[14] Fukushima Prefecture. Report of Full-scale Survey (Fourth Round Survey). 2021, https://www.pref.fukushima.lg.jp/uploaded/attachment/475143.pdf (in Japanese).

[15] Fukushima Prefecture. Report of survey at age 25 in Thyroid Ultrasound Examination. 2021, https://www.pref.fukushima.lg.jp/uploaded/attachment/475148.pdf (in Japanese).

[16] Nikiforov Y, Gnepp DR. Pediatric thyroid cancer after the Chernobyl disaster. Pathomorphologic study of 84 cases (1991–1992) from the Republic of Belarus. Cancer. 1994;74:748–766. 10.1002/1097-0142(19940715)74:2<748::AID-CNCR2820740231>3.0.CO;2-H 8033057

[17] Fukushima Medical University. Report of 3rd International Symposium of the Radiation Medical Science Center for the Fukushima Health Management Survey. 2021:[50–51 p.], http://kenko-kanri.jp/img/2021report_jp.pdf.

[18] Fukushima Prefecture. Result of each round of survey in the TUE program. 2021, https://www.pref.fukushima.lg.jp/uploaded/attachment/435495.pdf (in Japanese).

[19] Ohtsuru A, Midorikawa S, Ohira T, . Incidence of thyroid cancer among children and young adults in Fukushima, Japan, screened with 2 rounds of ultrasonography within 5 years of the 2011 Fukushima Daiichi Nuclear Power Station accident. JAMA Otolaryngol Head Neck Surg. 2019;145:4–11. 10.1001/jamaoto.2018.3121 30489622 PMC6439815

[20] Suzuki S, Suzuki S, Fukushima T, . Comprehensive survey results of childhood thyroid ultrasound examinations in Fukushima in the first four years after the Fukushima Daiichi Nuclear Power Plant accident. Thyroid. 2016;26:843–851. 10.1089/thy.2015.0564 27098220

[21] Nakaya T, Takahashi K, Takahashi H, . Spatial analysis of the geographical distribution of thyroid cancer cases from the first-round thyroid ultrasound examination in Fukushima Prefecture. Sci Rep. 2018;8:17661. 10.1038/s41598-018-35971-7 30518765 PMC6281575

[22] Ohira T, Takahashi H, Yasumura S, ; Fukushima Health Management Survey Group. Comparison of childhood thyroid cancer prevalence among 3 areas based on external radiation dose after the Fukushima Daiichi nuclear power plant accident: the Fukushima health management survey. Medicine (Baltimore). 2016;95:e4472. 10.1097/MD.0000000000004472 27583855 PMC5008539

[23] Ohira T, Takahashi H, Yasumura S, ; Fukushima Health Management Survey Group. Associations between childhood thyroid cancer and external radiation dose after the Fukushima Daiichi Nuclear Power Plant accident. Epidemiology. 2018;29:e32–e34. 10.1097/EDE.0000000000000839 29634592

[24] Ohira T, Ohtsuru A, Midorikawa S, ; Fukushima Health Management Survey group. External radiation dose, obesity, and risk of childhood thyroid cancer after the Fukushima Daiichi Nuclear Power Plant accident: the Fukushima Health Management Survey. Epidemiology. 2019;30:853–860. 10.1097/EDE.0000000000001058 31259849

[25] United Nations Scientific Committee on the Effects of Atomic Radiation. Attachment C-18: Estimated doses to evacuees in Japan for the first year. UNSCEAR 2013 Report, Annex A, Levels and effects of radiation exposure due to the nuclear accident after the 2011 great east-Japan earthquake and tsunami, Appendix C. 2014, https://www.unscear.org/docs/reports/2013/UNSCEAR_2013A_C-18_Doses_evacuees_Japan_first_year_2014-08.pdf.

[26] Ohira T, Shimura H, Hayashi F, ; Fukushima Health Management Survey Group. Absorbed radiation doses in the thyroid as estimated by UNSCEAR and subsequent risk of childhood thyroid cancer following the Great East Japan Earthquake. J Radiat Res. 2020;61:243–248. 10.1093/jrr/rrz104 32030428 PMC7246065

[27] United Nations Scientific Committee on the Effects of Atomic Radiation. Scientific Annex B, UNSCEAR 2020 Report, 2020, http://www.unscear.org/docs/publications/2020/UNSCEAR_2020_AnnexB_AdvanceCopy.pdf.

[28] Midorikawa S, Ohtsuru A, Murakami M, . Comparative analysis of the growth pattern of thyroid cancer in young patients screened by ultrasonography in Japan after a nuclear accident: the Fukushima Health Management Survey. JAMA Otolaryngol Head Neck Surg. 2018;144:57–63. 10.1001/jamaoto.2017.2133 29145557 PMC5833594

[29] Suzuki S, Nakamura I, Suzuki S, . Inappropriate suppression of thyrotropin concentrations in young patients with thyroid nodules including thyroid cancer: the Fukushima Health Management Survey. Thyroid. 2016;26:717–725. 10.1089/thy.2015.0499 26971545

[30] Fukushima T, Suzuki S, Ohira T, ; Thyroid Examination Unit of the Radiation Medical Center for the Fukushima Health Management Survey. Prevalence of ectopic intrathyroidal thymus in Japan: the Fukushima health management survey. Thyroid. 2015;25:534–537. 10.1089/thy.2014.0367 25778711

[31] Suzuki S, Midorikawa S, Fukushima T, ; Thyroid Examination Unit of the Radiation Medical Science Center for the Fukushima Health Management Survey. Systematic determination of thyroid volume by ultrasound examination from infancy to adolescence in Japan: the Fukushima Health Management Survey. Endocr J. 2015;62:261–268. 10.1507/endocrj.EJ14-0478 25735879

[32] Suzuki S, Midorikawa S, Matsuzuka T, . Prevalence and characterization of thyroid hemiagenesis in Japan: the Fukushima Health Management Survey. Thyroid. 2017;27:1011–1016. 10.1089/thy.2016.0662 28657504 PMC5564018

[33] Takahashi K, Takahashi H, Nakaya T, . Factors influencing the proportion of non-examinees in the Fukushima Health Management Survey for childhood and adolescent thyroid cancer: results from the baseline survey. J Epidemiol. 2020;30:301–308. 10.2188/jea.JE20180247 31204362 PMC7280055

[34] Fukushima Prefecture. Interrim summary of Fukushima Health Management Survey. 2016, https://www.pref.fukushima.lg.jp/uploaded/attachment/158522.pdf (in Japanese).

[35] Fukushima Prefecture. Summary of the Full-scale Survey (second-round survey) by the Subcommittee for TUE program. 2019, https://www.pref.fukushima.lg.jp/uploaded/attachment/351385.pdf (in Japanese).

[36] Shimura H, Suzuki S, Fukushima T, . Prevalence of thyroid nodular lesions in children and adolescents. Fukushima J Med Sci. 2014;60:196–202. 10.5387/fms.2014-28 25747610

[37] Taniguchi N, Hayashida N, Shimura H, ; Investigation Committee for the Proportion of Thyroid Ultrasound Findings. Ultrasonographic thyroid nodular findings in Japanese children. J Med Ultrason. 2013;40:219–224. 10.1007/s10396-013-0456-1 27277239

[38] Hayashida N, Imaizumi M, Shimura H, ; Investigation Committee for the Proportion of Thyroid Ultrasound Findings. Thyroid ultrasound findings in children from three Japanese prefectures: Aomori, Yamanashi and Nagasaki. PLoS One. 2013;8:e83220. 10.1371/journal.pone.0083220 24376666 PMC3871687

[39] Hayashida N, Imaizumi M, Shimura H, . Thyroid ultrasound findings in a follow-up survey of children from three Japanese prefectures: Aomori, Yamanashi, and Nagasaki. Sci Rep. 2015;5:9046. 10.1038/srep09046 25762224 PMC5390914

[40] Katanoda K, Kamo K, Tsugane S. Quantification of the increase in thyroid cancer prevalence in Fukushima after the nuclear disaster in 2011—a potential overdiagnosis? Jpn J Clin Oncol. 2016;46:284–286. 10.1093/jjco/hyv191 26755830 PMC4777612

[41] Rallison ML, Dobyns BM, Keating FR Jr, Rall JE, Tyler FH. Thyroid nodularity in children. JAMA. 1975;233:1069–1072. 10.1001/jama.1975.03260100039017 1174152

[42] Suzuki H, Uchida D, Sato T, Jyunma T, Yamada T, Nagao K. The significance of mass screening for thyroid diseases with palpation followed by ultrasonography to the university students. Campus Health. 2001;37:127–132 (in Japanese).

[43] Horiguchi K, Yoshida Y, Iwaku K, . Position paper from the Japan Thyroid Association task force on the management of low-risk papillary thyroid microcarcinoma (T1aN0M0) in adults. Endocr J. 2021;68(7):763–780. 10.1507/endocrj.EJ20-0692 33762511

[44] Welch HG, Black WC. Overdiagnosis in cancer. J Natl Cancer Inst. 2010;102:605–613. 10.1093/jnci/djq099 20413742

[45] Ito Y, Onoda N, Okamoto T. The revised clinical practice guidelines on the management of thyroid tumors by the Japan Associations of Endocrine Surgeons: core questions and recommendations for treatments of thyroid cancer. Endocr J. 2020;67:669–717. 10.1507/endocrj.EJ20-0025 32269182

[46] Diagnostic Criteria Committee on Thyroid Ultrasonography in Japan Association of Thyroid and Breast Sonology. Diagnostic procedure for thyroid nodular lesions. In: Japan Association of Thyroid and Breast Sonology, editor. *Thyroid Ultrasound Guidebook for Thyroid Gland (2nd Edition)*. Tokyo: Nankodo Co., Ltd.; 2012:28–29 (in Japanese).

[47] Committee on Guideline for Clinical Practice for the Management of Thyroid Nodules. Fine needle aspiration cytology. In: The Japan Thyroid Association, editor. *Guideline for Clinical Practice for the Management of Thyroid Nodules in Japan*. Tokyo: Nankodo Co., Ltd.; 2013:59–64 (in Japanese).

[48] Diagnostic Criteria Committee on Thyroid Ultrasonography in Japan Association of Thyroid and Breast Sonology. Diagnostic procedure for thyroid nodular lesions. In: Japan Association of Thyroid and Breast Sonology, editor. *Thyroid Ultrasound Guidebook for Thyroid Gland (3rd Edition)*. Tokyo: Nankodo Co., Ltd.; 2016:48–53 (in Japanese).

[49] The Japanese Society of Thyroid Surgery and The Japan Society of Endocrine Surgeons. *Guidelines for the management of thyroid tumors*. Tokyo. Kanehara & Co., Ltd.; 2010 (in Japanese).

[50] The JAES and JSTS Task Force on the Guideline on Management of Thyroid Tumors. Clinical practice guidelines on the management of thyroid tumors. J JAES JSTS (Nihon Naibunpitu Kojosen Geka Gakkai Zasshi). 2018;35(Supple 3):1–87.

[51] Haugen BR, Alexander EK, Bible KC, . 2015 American Thyroid Association management guidelines for adult patients with thyroid nodules and differentiated thyroid cancer: the American Thyroid Association guidelines task force on thyroid nodules and differentiated thyroid cancer. Thyroid. 2016;26:1–133. 10.1089/thy.2015.0020 26462967 PMC4739132

[52] Haddad RI, Nasr C, Bischoff L, . NCCN Guidelines Insights: Thyroid Carcinoma, Version 2.2018. J Natl Compr Canc Netw. 2018;16:1429–1440. 10.6004/jnccn.2018.0089 30545990

[53] Tessler FN, Middleton WD, Grant EG, . ACR Thyroid Imaging, Reporting and Data System (TI-RADS): White Paper of the ACR TI-RADS Committee. J Am Coll Radiol. 2017;14:587–595. 10.1016/j.jacr.2017.01.046 28372962

[54] Filetti S, Durante C, Hartl D, ; ESMO Guidelines Committee. Thyroid cancer: ESMO Clinical Practice Guidelines for diagnosis, treatment and follow-up. Ann Oncol. 2019;30:1856–1883. 10.1093/annonc/mdz400 31549998

[55] Russ G, Bonnema SJ, Erdogan MF, Durante C, Ngu R, Leenhardt L. European Thyroid Association guidelines for ultrasound malignancy risk stratification of thyroid nodules in adults: the EU-TIRADS. Eur Thyroid J. 2017;6:225–237. 10.1159/000478927 29167761 PMC5652895

[56] Shimura H, Yokoya S, Kamiya K. An Accurate Picture of Fukushima’s Thyroid Ultrasound Examination Program. Arch Pathol Lab Med. 2020;144:797. 10.5858/arpa.2019-0465-LE 33761528

[57] Shimura H, Matsuzuka T, Suzuki S, . Fine needle aspiration cytology implementation and malignancy rates in children and adolescents based on Japanese guidelines: the Fukushima Health Management Survey. Thyroid. 2021;31:1683–1692. 10.1089/thy.2021.0072 34762538

[58] Takahashi H, Takahashi K, Shimura H, . Simulation of expected childhood and adolescent thyroid cancer cases in Japan using a cancer-progression model based on the National Cancer Registry: application to the first-round thyroid examination of the Fukushima Health Management Survey. Medicine (Baltimore). 2017;96:e8631. 10.1097/MD.0000000000008631 29310337 PMC5728738

[59] Suzuki S, Bogdanova TI, Saenko VA, . Histopathological analysis of papillary thyroid carcinoma detected during ultrasound screening examinations in Fukushima. Cancer Sci. 2019;110:817–827. 10.1111/cas.13912 30548366 PMC6361578

[60] Yokoya S, Iwadate M, Shimura H, . Investigation of thyroid cancer cases that were not detected in the Thyroid Ultrasound Examination program of the Fukushima Health Management Survey but diagnosed at Fukushima Medical University Hospital. Fukushima J Med Sci. 2020;65:122–127. 10.5387/fms.2019-26 31839647 PMC7012588

[61] Suzuki S. The features of childhood and adolescent thyroid cancer after the Fukushima Nuclear Power Plant accident. *Thyroid Cancer and Nuclear Accidents: Long-Term Aftereffects of Chernobyl and Fukushima*. Elsevier; 2017:155–163.

[62] Demidchik YE, Demidchik EP, Reiners C, . Comprehensive clinical assessment of 740 cases of surgically treated thyroid cancer in children of Belarus. Ann Surg. 2006;243:525–532. 10.1097/01.sla.0000205977.74806.0b 16552205 PMC1448966

[63] Nikiforov YE. Radiation-induced thyroid cancer: What we have learned from Chernobyl. Endocr Pathol. 2006;17:307–317. 10.1007/s12022-006-0001-5 17525478

[64] Iwadate M, Mitsutake N, Matsuse M, . The clinicopathological results of thyroid cancer with BRAFV600E mutation in the young population of Fukushima. J Clin Endocrinol Metab. 2020;105:e4328–e4336. 10.1210/clinem/dgaa573 32827026

[65] Klugbauer S, Lengfelder E, Demidchik EP, Rabes HM. High prevalence of RET rearrangement in thyroid tumors of children from Belarus after the Chernobyl reactor accident. Oncogene. 1995;11:2459–2467. 8545102

[66] Thomas GA, Bunnell H, Cook HA, . High prevalence of RET/PTC rearrangements in Ukrainian and Belarussian post-Chernobyl thyroid papillary carcinomas: a strong correlation between RET/PTC3 and the solid-follicular variant. J Clin Endocrinol Metab. 1999;84:4232–4238. 10.1210/jcem.84.11.6129 10566678

[67] Kazakov VS, Demidchik EP, Astakhova LN. Thyroid cancer after Chernobyl. Nature. 1992;359:21. 10.1038/359021a0 1522879

[68] Demidchik YE, Saenko VA, Yamashita S. Childhood thyroid cancer in Belarus, Russia, and Ukraine after Chernobyl and at present. Arq Bras Endocrinol Metabol. 2007;51:748–762. 10.1590/S0004-27302007000500012 17891238

[69] Cardis E, Kesminiene A, Ivanov V, . Risk of thyroid cancer after exposure to 131I in childhood. J Natl Cancer Inst. 2005;97:724–732. 10.1093/jnci/dji129 15900042

[70] Zablotska LB, Ron E, Rozhko AV, . Thyroid cancer risk in Belarus among children and adolescents exposed to radioiodine after the Chornobyl accident. Br J Cancer. 2011;104:181–187. 10.1038/sj.bjc.6605967 21102590 PMC3039791

[71] Brenner AV, Tronko MD, Hatch M, . I-131 dose response for incident thyroid cancers in Ukraine related to the Chornobyl accident. Environ Health Perspect. 2011;119:933–939. 10.1289/ehp.1002674 21406336 PMC3222994

