# Supplementary material for: A Comprehensive Review of the Progress and Evaluation of the Thyroid Ultrasound Examination Program, the Fukushima Health Management Survey
Source: J Epidemiol. 2022 Dec 5;32(Suppl 12):S23–35. doi: 10.2188/jea.JE20210271 (PMC9703930; doi:10.2188/jea.JE20210271)
Supplement: Supplementary file 1 [file je-32-S023-s001.pdf]

eMaterial 1. Supplemental methods

Sonographic features for the diagnosis of thyroid nodules

In 2011, The Japan Society of Ultrasonics in Medicine (JSUM) published ultrasound diagnostic criteria for thyroid nodules.[1, 2] To establish these criteria, a multi-center study was performed and revealed that the shape, border character, and internal echo level showed the highest sensitivity and specificity, followed by the edge definition and heterogeneity of internal echoes.[3] These characteristics were selected as the primary findings. In addition, the marginal hypoechoic zone and fine strong echoes were considered secondary findings[1] because the statistical differences between benign and malignant nodules were lower than those in the primary findings.[3]

Risk assessment of thyroid carcinoma by ultrasound findings

Recently, in Japan, it has been reported that adult cases with papillary thyroid microcarcinoma 10 mm or less without metastasis or extrathyroidal invasion for the recurrent nerve or trachea have less tumor growth and good prognosis.[4] Therefore, even in microcarcinomas of 10 mm or less, detailed ultrasonography may contribute to reducing the risk of overdiagnosis of thyroid cancer by detailed assessment of risk. In a multivariate study of risk factors for tumor growth, macroscopic (coarse) calcification (>1 mm), agglutinated calcification with acoustic shadow, rim calcification completely aligned along the rim of the tumors, and poorer vascularity were significantly correlated with non-progression, and extrathyroidal invasion, lymph node metastasis, and poorly differentiated composition were significantly associated with adverse outcomes.[5, 6] In patients with surgically treated micropapillary carcinoma, ill-defined tumor edges and

fine strong echoes on ultrasound images have been shown to be associated with recurrence and lateral lymph node metastasis of thyroid cancer.[7] In addition, risk factors for lymph node metastasis of microcarcinoma were shown to be male sex, younger age (<45 years), larger tumor size, microcalcification, extrathyroidal invasion, hypoechogenicity within the tumor, and multicentricity.[8-14] The line of evidence has led to the publication of fine needle aspiration cytology (FNAC) implementation criteria based on risk assessment by ultrasonographic findings in Japan and subsequently worldwide.[2]

Implementation criteria for fine needle aspiration cytology

The Japan Association of Breast and Thyroid Sonology (JABTS) published criteria for the management of thyroid nodules and presented a diagnostic flowchart for nodular thyroid disease to provide a standard.[2, 15] This flowchart categorizes nodular lesions into solid and cystic nodules, providing the criteria for each of them. In 2016, the JABTS revised the guidebook (3rd edition), and only the criteria for the management of cystic nodules were revised.[2, 16]

According to this criterion, FNAC is recommended for nodules measuring 5.1–10.0 mm in diameter that are strongly suspicious for thyroid carcinoma according to the primary and secondary findings in JSUM diagnostic criteria; that is, when most of the malignant sonographic findings are observed.[2, 15] FNAC is also recommended for nodules sized 10.1–20.0 mm in diameter that are suspicious for carcinoma according to the above criteria (ie, when some of the malignant sonographic findings were observed). All nodules >20 mm in diameter and nodules showing spongiform patterns are subjected to FNAC.

If cystic lesions are observed, they are classified according to the presence or absence of a solid portion. If there is no solid component, FNAC is not recommended for ≤20.0 mm, and aspiration of the cyst fluid is considered for cysts of 20.1 mm or greater in diameter to relieve symptoms.[15, 16] The ratio of the solid component in the maximum cross-section is divided into 50% or more and less than 50%, and the former follows the above-mentioned management criterion for solid nodules.[2, 16] In the latter case, FNAC is not recommended when the maximum diameter, including the cystic component, is 5 mm or less. For nodules with a maximum diameter of ≥5.1 mm and ≤20.0 mm, FNAC is recommended when a nodule is suspected to have extramural infiltration, even if the solid component is less than 5.1 mm. FNAC is recommended when there are multiple malignant findings among the irregular surfaces of solid components, fine multiple high-echo spots, and increased blood flow for a nodule with 5.1 to 10.0 mm in diameter of a solid component. For a nodule with a solid area exceeding 10.0 mm, FNAC is recommended when any of the above malignant findings are present. If the maximum diameter of a cystic nodule is 20.1 mm or more, in principle, FNAC is performed.

REFERENCES

1. Kitaoka M, Miyamoto Y, Fukunari N, et al. Ultrasound diagnostic criteria for thyroid nodule. Jpn J Med Ultrasonics. 2011;36:669-670.
2. Shimura H, Matsumoto Y, Murakami T, Fukunari N, Kitaoka M, Suzuki S. Diagnostic Strategies for Thyroid Nodules Based on Ultrasonographic Findings in Japan. Cancers (Basel). 2021;13(18):4629.
3. Shimura H, Haraguchi K, Hiejima Y, et al. Distinct diagnostic criteria for ultrasonographic examination of papillary thyroid carcinoma: a multicenter study. Thyroid. 2005;15:251-258.
4. Horiguchi K, Yoshida Y, Iwaku K, et al. Position paper from the Japan Thyroid Association task force on the management of low-risk papillary thyroid microcarcinoma (T1aN0M0) in adults. Endocr J. 2021;68(7):763-780.
5. Sugitani I, Toda K, Yamada K, Yamamoto N, Ikenaga M, Fujimoto Y. Three distinctly different kinds of papillary thyroid microcarcinoma should be recognized: our treatment strategies and outcomes. World J Surg. 2010;34:1222-1231.
6. Fukuoka O, Sugitani I, Ebina A, Toda K, Kawabata K, Yamada K. Natural History of Asymptomatic Papillary Thyroid Microcarcinoma: Time-Dependent Changes in Calcification and Vascularity During Active Surveillance. World J Surg. 2016;40:529-537.
7. Ito Y, Kobayashi K, Tomoda C, et al. Ill-defined edge on ultrasonographic examination can be a marker of aggressive characteristic of papillary thyroid microcarcinoma. World J Surg. 2005;29:1007-1011; discussion 1011-1002.
8. Zhao C, Jiang W, Gao Y, Niu W, Zhang X, Xin L. Risk factors for lymph node metastasis (LNM) in patients with papillary thyroid microcarcinoma (PTMC): role of preoperative ultrasound. J Int Med Res. 2017;45:1221-1230.
9. Xu Y, Xu L, Wang J. Clinical predictors of lymph node metastasis and survival rate in papillary thyroid microcarcinoma: analysis of 3607 patients at a single institution. J Surg Res. 2018;221:128-134.
10. Huang XP, Ye TT, Zhang L, et al. Sonographic features of papillary thyroid microcarcinoma predicting high-volume central neck lymph node metastasis. Surg Oncol. 2018;27:172-176.
11. Wang WH, Xu SY, Zhan WW. Clinicopathologic Factors and Thyroid Nodule Sonographic Features for Predicting Central Lymph Node Metastasis in Papillary Thyroid Microcarcinoma: A Retrospective Study of 1204 Patients. J Ultrasound Med. 2016;35:2475-2481.
12. Gao Y, Qu N, Zhang L, Chen JY, Ji QH. Preoperative ultrasonography and serum thyroid-

stimulating hormone on predicting central lymph node metastasis in thyroid nodules as or suspicious for papillary thyroid microcarcinoma. Tumour Biol. 2016;37:7453-7459.

13. Lee YS, Lim YS, Lee JC, et al. Ultrasonographic findings relating to lymph node metastasis in single micropapillary thyroid cancer. World J Surg Oncol. 2014;12:273.

14. Kim KE, Kim EK, Yoon JH, Han KH, Moon HJ, Kwak JY. Preoperative prediction of central lymph node metastasis in thyroid papillary microcarcinoma using clinicopathologic and sonographic features. World J Surg. 2013;37:385-391.

15. Diagnostic Criteria Committee on Thyroid Ultrasonography in Japan Association of Thyroid and Breast Sonology. Diagnostic procedure for thyroid nodular lesions. In: Japan Association of Thyroid and Breast Sonology, editor. Thyroid Ultrasound Guidebook for Thyroid Gland (2nd Edition). Tokyo: Nankodo Co., Ltd.; 2012. p. 28-29.

16. Diagnostic Criteria Committee on Thyroid Ultrasonography in Japan Association of Thyroid and Breast Sonology. Diagnostic procedure for thyroid nodular lesions. In: Japan Association of Thyroid and Breast Sonology, editor. Thyroid Ultrasound Guidebook for Thyroid Gland (3rd Edition). Tokyo: Nankodo Co., Ltd.; 2016. p. 48-53.
